# ﻿A new species of *Encelia* (Compositae, Heliantheae, Enceliinae) from the southern Baja California Peninsula

**DOI:** 10.3897/phytokeys.212.91190

**Published:** 2022-11-04

**Authors:** Jose Luis Leon-De La Luz, Isaac H. Lichter-Marck

**Affiliations:** 1 Programa de Protección Ambiental y Cambio Global, Centro de Investigaciones Biológicas de Noreste, Apdo. Postal 128, La Paz, Baja California Sur, 23000 Mexico Programa de Protección Ambiental y Cambio Global, Centro de Investigaciones Biológicas de Noreste La Paz Mexico; 2 Department of Integrative Biology & Jepson Herbarium, University of California Berkeley, Berkeley, California, USA University of California Berkeley Berkeley United States of America; 3 Department of Ecology and Evolutionary Biology, University of California Los Angeles, Los Angeles, California, USA University of California Los Angeles Los Angeles United States of America

**Keywords:** Asteraceae, Cape region, DNA barcoding, narrow endemism, synantherology, taxonomy

## Abstract

Here, we describe and illustrate *Enceliabalandra***sp. nov.**, a new species of Compositae from the Baja California Peninsula. It is rare and known only from the rocky hills around Puerto Balandra and Pichilingüe, inside the bay of La Paz, in the State of Baja California Sur, Mexico. We determine that this new species has affinities with *Encelia*, based on its suffruticose woody habit, neuter ray florets and compressed disc cypselae with a cleft apex. The taxonomic placement within *Encelia* is supported by nuclear ribosomal sequence data from two regions, ITS and ETS. We also present detailed photographs, a conservation assessment and a dichotomous key to the *Encelia* of the southern Baja California Peninsula. Finally, we discuss the uniqueness of *Enceliabalandra* amongst peninsular *Encelia* and its potential significance for understanding the enigmatic biogeography of this ecologically important genus.

## Abstract (es_mx)

Se ilustra y describe a una nueva especie de Asteraceae, *Enceliabalandra*. Se conoce solo de las laderas rocosas de los cerros próximos a puerto Balandra y Pichilingue, dentro de la bahía de La Paz, en Baja California Sur, México. Encontramos que esta nueva especie tiene ciertas semejanzas con otras de *Encelia* por su hábito semi-arbustivo, las flores radiales neutras y cipselas comprimidas con ápice hendido. Confirmamos tal condición con datos de secuencia para las regiones ITS y ETS del genoma. También presentamos fotografías detalladas, un evaluación de conservación, y un clave dichotomus para los *Encelia* de Baja California sur. Se discute la semejanza con las especies peninsulares más cercanas de *Encelia*, se presenta una clave dicotómica para los taxones australes de la península de Baja California, y finalmente se muestran imágenes detalladas de esta nueva especie.

## ﻿Introduction

*Encelia* Adans is a genus of New World sunflowers nested within tribe *Heliantheae*, *sensu*Panero and Funk (2002), based on the presence of paleae, agamous ray florets and obcompressed cypselae with flattened margins. The genus includes shrubs, suffrutescent perennials and few herbaceous perennials that are a dominant and ecologically important element in arid environments in the southwest U.S. and northwest Mexico, as well as western South America. The genus was most recently treated by Clark (1998) who cited 17 species and seven subspecies within two subclades, the *Californica* and *Frutescens* groups. These subgeneric groups were supported with molecular phylogenetic data by Fehlberg and Ranker (2007), who included all 20 minimum-rank taxa of *Encelia* in their study of subtribe *Enceliinae* Panero, encompassing the additional genera, *Enceliopsis* (A. Gray) A. Nelson, *Flourensia* DC. and *Geraea* Torr. & A. Gray. Recently, a phylogeny, based on next generation sequencing (RADseq) data by Singhal et al. (2021), has resolved species level relationships within *Encelia* and provided support for the *Californica* and *Frutescens* groups with the exceptions of two narrow endemics in the Baja California Peninsula, *E.densifolia* C. Clark & Kyhos and *E.ravenii* Wiggins, which form a previously undetected early-diverging clade.

The Baja California Peninsula harbours the majority of species diversity in *Encelia*, with nine taxa that fully, or partially, overlap in geographical distribution. The two major subclades proposed by Clark (1998), the *Californica* and *Frutescens* groups, contain five and four taxa on the Peninsula, respectively. According to Rebman et al. (2016) and distribution maps in Clark (2000), three taxa of *Encelia* are found in the Cape region of Baja California Sur: E.farinosaA. Gray ex Torreyvar.radians Brandegee ex S.F. Blake (362: 1913), E.farinosaA. Gray ex Torreyvar.phenicodonta (S.F. Blake) I.M. Johnston (1198: 1924) and *E.palmeri* Vasey & Rose (535: 1889). E.farinosavar.radians is found in the lowlands of the eastern Cape region, while E.farinosavar.phenicodonta and *E.palmeri* reach their southernmost extent in the Cape region, but are more common in desert habitats in the northern peninsula. An additional taxon, *E.conspersa* Benth. (26: 1844) is an endemic of Magdalena Bay, close to the Cape region.

The Balandra/Pichilingue area is an important recreation destination for residents and visitors of the City of La Paz, in the State of Baja California Sur (BCS), Mexico. A relatively small area of 2,512 hectares were decreed in 2012 as a combined marine and terrestrial area, or Zona de Protección de Flora y Fauna (ZPFF) by the Comisión Nacional de Areas Naturales Protegidas (CONANP) of Mexico. The area encompasses a coastal wetland, composed mainly of intertidal habitats, sand dunes, mangroves and xeric shrubland on the slopes of low mountains and hills. In September 2014, the first author was asked to make a floristic checklist of the ca. 1,000 hectares of terrestrial surface in the Balandra/Pichilingue area as part of an integrated management plan for the protected area. One plant observed during this survey was an uncommon suffrutescent, low perennial herb, in vegetative stage, with characteristics of the Heliantheae alliance of the sunflower family (Compositae). Based on limited sampling of mostly vegetative material, we were able to discern basic characters, such as solitary, terminal capitula on long, scape-like peduncles and receptacular bracts, which led the plant to be determined as *Heliopsis* Pers. in the checklist, following the taxonomic key in Wiggins (1980).

Heavy rainfall in the early winter of 2019–2020 brought favourable conditions to the Balandra/ Pichilingue area, allowing us to collect suitable material, photograph the plant in flower and fruit and determine the genus of the plant with confidence using dichotomous keys (Shreve and Wiggins 1964; Wiggins 1980) and, later, to sequence DNA from two regions of the nuclear ribosomal cistron, the Internal Transcribed Spacer (ITS) and External Transcribed Spacer (ETS). This approach allowed us to present both morphological and molecular phylogenetic evidence that this new plant is a previously undescribed member of the genus *Encelia*, which we describe and illustrate here.

## ﻿Materials and methods

### ﻿Study site

**Figure 1.**
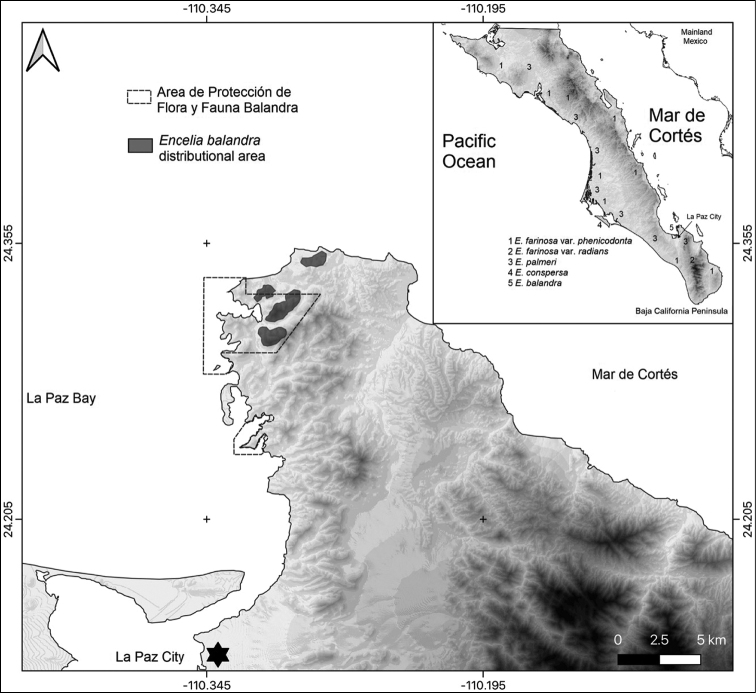
Range map of *Enceliabalandra*. Geographical location of the study site in Baja California Sur (BCS), Mexico illustrating the Balandra/Pichilingüe Protected Area with a dashed polygon. The dark-grey area marks the zone where 17 plants were located and collected. The light-grey tone shows mountainous terrain. Numbers (1–5) show the distribution of the southern *Encelia* taxa in Baja California Sur, Mexico.

Biogeographically, the Balandra or Pichilingue Hills constitute a disjunct fragment of the Sonoran Desert Province (*sensu*Shreve and Wiggins 1964) that falls within the Central Gulf Coast sub-province (Rebman et al. 2016). This region forms a long strip of lowlands along the east coast of most of the Baja California Peninsula, under the environmental influence of the Gulf of California. The Balandra/Pichilingue Hills form the southern point of the great Bay of La Paz. Geologically, the rocks are part of the complex Comondú Formation (Aranda-Gómez and Pérez-Venzor 1988), whose orogenesis occurred during the early Miocene age, these volcanic mountains representing the backbone of the Baja California Peninsula (see Fig. 1 for the location of the study area, including the distributional range of *Encelia* spp. in BCS).

### ﻿Field collection

The exceptional rainy season of winter 2019–2020 made it possible to locate additional individuals of the focal plant in the Balandra Hills. Three populations were located, separated by approximately 800 m, at el Tecolote Beach, the hills of Rancho San Lorenzo and Balandra Beach. Reviewing the collections of undetermined Compositae at HCIB Herbarium (abbreviations following Thiers 2020) revealed two additional exemplars of this same plant previously collected within the Balandra Protected Area in 1994 and 1995. To our knowledge, specimens of this new taxon have not yet been accessioned at other herbaria.

### ﻿Morphological study

A careful examination of morphological characters was carried out based on the samples of the vegetative and reproductive material. Morphology was compared with images of all currently recognised minimum rank taxa of *Encelia* of the Baja California Peninsula and western Mexico in several taxonomic treatments (Blake 1913; Shreve and Wiggins 1964; Wiggins 1980; Clark 1998), as well online image databases (Clark 2000; NaturaLista 2020; BajaFlora 2021). Measurements of vegetative and reproductive structures were made in the field, as well as on rehydrated herbarium material. Photographs were taken using a Stereo Stemi DV4 Spot microscope (Zeiss, Jena, Germany), a Coolpix B500 digital camera (Nikon, Minato City, Tokyo, Japan), and a Tough TG-5 digital camera (Olympus, Shinjuku City, Japan).

### ﻿DNA extraction, amplification and sequencing

Total genomic DNA was obtained from 3–5 silica-dried leaves using a DNeasy plant mini-kit (Qiagen, inc., Valencia, California) following the protocol recommended by Qiagen. To counteract the PCR prohibitive effects of co-precipitated polyasaccharides present in some Asteraceae, genomic DNA was diluted to 1:50 parts with nuclease free water. Amplification and sequencing of the Internal Transcribed Spacer (ITS) region was achieved using primers ITS4 and ITS5 (White et al. 1990). The External Transcribed Spacer (ETS) was amplified and sequenced using primers 18S-ETS (Markos and Baldwin 2001) and ETS-HEL-1 (Baldwin and Markos 1998). Both ITS and ETS were amplified with Accupower PCR premix (Bioneer, Daejeon, South Korea) and 17 µl of 1:50 diluted DNA. PCR conditions using the MJ PCT-100 thermalcycler (Marshall scientific, Hampton, NH) replicating the protocol developed by Fehlberg and Ranker (2007) for *Encelia* as follows: 97 °C for 1 min; 40 cycles of 97 °C for 10 sec, 48 °C for 30 sec, 72 °C for 20 sec with an additional 4 sec per cycle; and 72 °C for 7 min. Post-PCR products were cleaned using magnetic SPRA beads and forward and reverse reads of all loci obtained using Sanger sequencing at the Barker Hall sequencing facility at UC Berkeley. Sequences were assembled and edited in sequencer v. 4.2 (Gene codes corp, Ann Arbor, Michigan). After a query of publicly available sequence data using nucleotide BLAST confirmed a close match to previously sequenced members of *Encelia*, we aligned sequences visually with the ITS and ETS alignments of Fehlberg and Ranker (2007) for tribe Enceliinae in the software programme Aliview (Larsson 2014). A Maximum Likelihood (ML) phylogenetic tree based on a concatenated matrix of ITS and ETS loci was inferred using RAxML (Stamatakis 2014) on the CIPRES computing portal using 1000 boot strap replicates and a GTRCAT molecular substitution model and visualised in the programme FigTree.

## ﻿Results

### ﻿ Encelia balandra

Taxon classificationPlantaeAsteralesCompositae

León De La Luz & Lichter-Marck sp. nov.

6E85C95B-A720-5431-AA95-87AD7CEFF030

urn:lsid:ipni.org:names:77307631-1

Figs 2 , 3

#### Remark.

Capitulum anatomy, particularly solitary heads erect in fruiting, places this new species with members of the frutescens clade, such as *E.frutescens* and *E.virginensis*. Molecular analysis also places the species in alliance with the Frutescens clade. The combination of glabrous, epappose cypselae, strigose hairs, a suffruticose habit and solitary heads are unique to this species.

**Figure 2.**
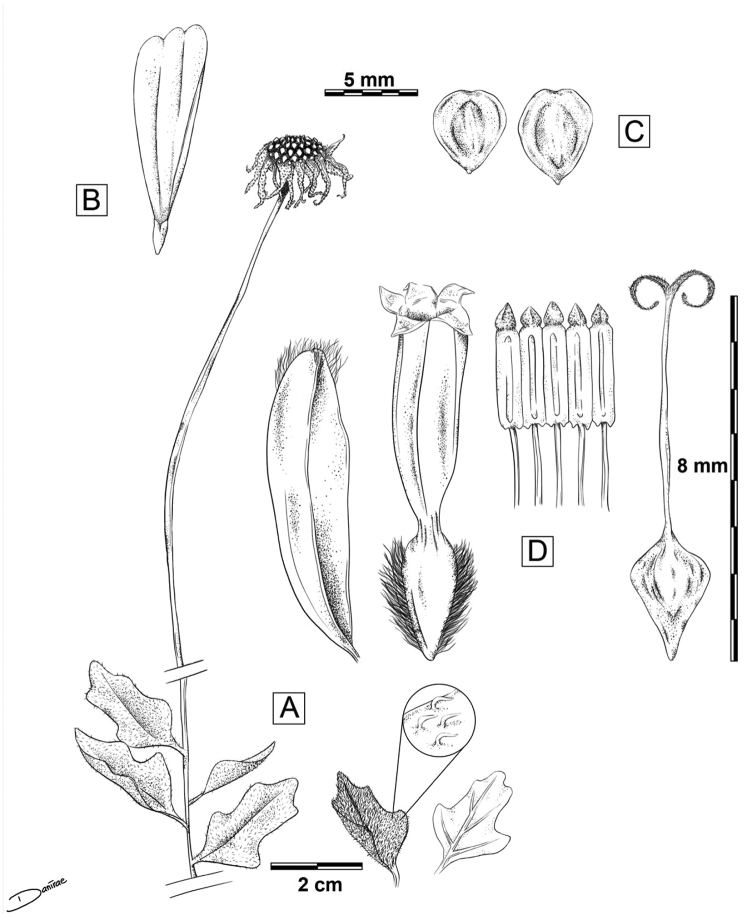
Line drawings of *Enceliabalandra* sp. nov. **A** mature branch with basal leaves and terminal peduncle **B** strigulose indumentum on leaves, also present in peduncles and phyllaries; also, leaf venation pattern **C** ray floret, neuter **D** cypsela, note a broad shallow apical cleft and glabrous faces, epappose **E** palea **F** disc floret showing silky villous margins of immature achene **G** anther cylinder, opened out **H** immature achene/cypsela and style. Art by Danira León C, based on material from JLLL 12900.

#### Description.

Deciduous perennial herb, suffrutescent, up to 40 cm high, with dense foliage when present. **Leaves** entire, fleshy, alternate, (2–)3 cm long × 1.5(–2) cm wide, ovate-lanceolate in outline, margins crenate, often with 4 conspicuous, symmetric lobes, basal pair larger than distal, petiole short 2–3 mm long, venation supra-basal and imperfect with 1 or 2 pairs of veins, apex acute, indumentum strigulose, eglandular, multicellular trichomes 1 mm, adpressed, with a semi-bulbose base. Solitary **Inflorescence**, capitula terminal, peduncles naked, 14–20 cm long in bloom, up to 25 cm in fruit. **Capitula** 2.5–3 cm diameter, heterogamous, radiate; **involucre** 1.5–2 cm diameter, phyllaries tri-seriate, 6–9 bracts per series, bracts herbaceous lanceolate to ovate-lanceolate, inner slightly larger than outer, inner 7–8 mm long × 2 mm wide, outer 6–7 mm long × 2 mm wide, occasionally villous at the apex, barely connate at base, scarious to touch, persistent after fruiting; **receptacle** chaffy, slightly convex, 13–15 mm diameter, 4–6 mm high, some paleaes empty < 1 mm long, silky villous; **paleaes** subtending disc florets, scarious, concave, subulate, acrescent in age, up to 5 mm long × 3 mm wide, barely villous at ends. **Ray florets** neuter, 12–15, uniseriate, ray limb yellow, ± spathulate to oblong-elliptic in outline, 10(–12) mm long × 6 mm wide, apex 2-toothed, tube 2 mm long. **Disc florets** perfect, but either hermaphroditic or functionally male, 40–50+, corolla actinomorphic with narrow cylindrical tube 6 mm long, throat cylindrical-funnelform 1 mm long × 1 mm wide; corolla lobes 5, acute, dark in colour, reflexed, tiny oil dots sparse in the inner side; style 5–6 mm long, stigma branches coiled, linear subulate ± 1.5 mm long, surpassing corolla lobes, short pubescent and papillate outside; stamens 5, 4 mm long, surpassing corolla lobes, but not stigma branches, with stiff rhombic-shaped terminal appendage at level of corolla lobes, thinly glandular, sub-auricular at base, filaments distinct, ±2 mm long. **Cypselae** monomorphic (but some larger than others), 4–5 mm long × 4(-3) mm wide, laterally compressed, obovoid in outline, with a broad shallow apical cleft, margins densely silky villous (immature), at maturity margins with a thin chartaceous edge < 1 mm, faces glabrous, black, smooth in texture, epappose.

**Figure 3.**
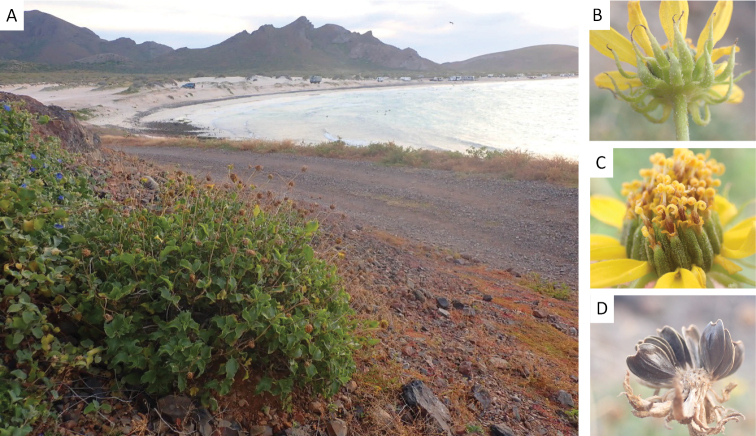
Photographs of *Enceliabalandra* illustrating habitat and morphological characters of the capitula and cypselae **A** habitat on the rocky hills north of Playa Balandra **B** abaxial view of capitulum showing phyllaries **C** adaxial view of capitulum showing ray limbs and paleae enfolding disc florets **D** cypselae. Photos by ILM.

#### Type.

Mexico: Baja California Sur: municipio de La Paz, zona de Protección de Flora y Fauna Balandra, colina adyacente al estacionamiento del Balneario Balandra. 24.324894°N, -110.326251°W, ca. 60 m de elevación, laderas rocosas, 19 de Enero 2020, J.L. León-de la Luz 13007 (holotype: HCIB 31869, isotypes to be distributed UC, MEXU, SD).

***Paratypes*** – Mexico. Baja California Sur: Municipio de La Paz, cerca El Tecolote 6 km al N de Puerto Pichilingue. 24.2000°N, -110.23300°W, ca. 7 m de elevación, 2 de Septiembre 1994, M. Domínguez León 762, HCIB 4740. Municipio de La Paz, Cerro Balandra, 2 km al N de Puerto Pichilingue. 24.323500°N, -110.326700°W, 18 m de elevación, 20 de Enero 1995, M. Domínguez León 959, HCIB 4739. Municipio de La Paz, ladera rocosa cerca de El Pulgero. 24.346067°N, -110.270051°W, 8 m de elevación, 20 de Enero 1995, J.L. León de la Luz 7517, HCIB 5127. Bahía de La Paz, Sierra Riolítica, Cerro Manglar El Merito. 24.301191°N, -110.324712°W, 28 m de elevación, 22 de Noviembre 2013, J.L. León de la Luz 11891, HCIB 624. Bahía de La Paz, Zona de Protección de Flora y Fauna Balandra, Cerro adjunto al Tecolote. 24.3406634°N, -110.304868°W, 15 m de elevación, 10 de Octubre 2019, J.L. León de la Luz 12900, HCIB 31868. Municipio de La Paz, Playa El Tecolote, cerrito al extremo Este de la playa. 24.341095°N, -110.304525°W, 16 m de elevación, 1 de Octubre 2022, J. L. León de la Luz 13125.

#### Etymology.

Balandra Beach is an emblematic place near La Paz, the capitol city of Baja California Sur, which is considered by many to be one of the most scenic beaches in all of Mexico.

#### Distribution and ecology.

This species is known only from the hills of the Balandra/Pichilingue area, where a total of 20 documented individuals occur over an area of no more than 500 hectares. *E.balandra* grows on coarse gravelly soils to bare rocky outcrops on the slopes of the hills. Some plants were documented on gravelly soil in the immediate vicinity of the seashore. Some insect visitors observed actively pollinating the plants were bees (Apidae), hoverflies (Syrphidae) and wasps (Hymenoptera).

#### Conservation status.

*E.balandra* is a new species described from an area that is an important touristic destination. The area currently faces pressure due the growing influx of local and international visitors, which spend time either at the beaches or hiking in the hills. Thus far, 20 individuals of *E.balandra* have been found. Taxonomic resolution of this new species lays the foundation for more targeted surveys to illuminate its population status, environmental restrictions and threats from anthropogenic pressures. Until more information about its status is collected, *E.balandra* should be categorised as data deficient (DD) under the IUCN current guidelines for Categories and Criteria (IUCN Standards and Petitions Committee 2022). However, we expect that, once surveyed, this rare plant would qualify as having a very restricted distribution with high plausibility of being threatened with extinction (VU). More information and material should be gathered and the environmental authority of Mexico (SEMARNAT) should consider managing visitation within the immediate area as a precautionary measure.

#### Phenology.

*E.balandra* is herbaceous with a woody taproot. Leaves appear to grow opportunistically in response to late summer, autumn or early winter rainfall. The peduncle and its capitulum, or head, reach anthesis some 20–30 days after a heavy rainfall, when foliar growth ceases. Some inflorescences and fruits could be present in the early autumn after summer rainstorms, but vigorous flowering also occurs if enough early winter rain is present.

#### Evolutionary affinities.

**Figure 4.**
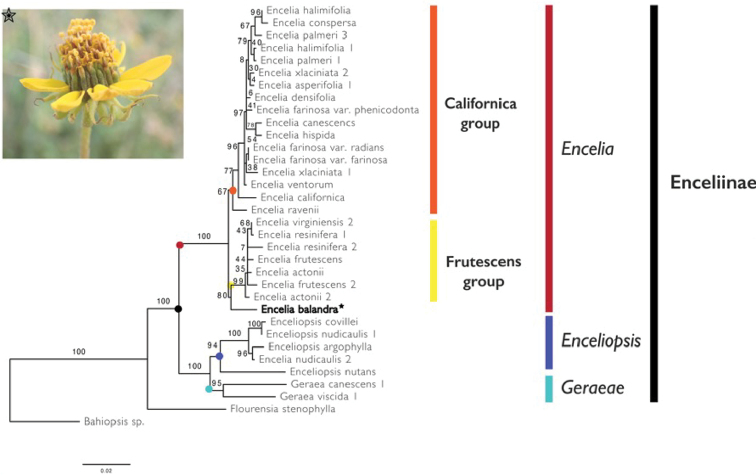
Phylogenetic position of *Enceliabalandra*. Maximum Likelihood phylogeny of subtribe Enceliinae, based on a combined data matrix of Internal Transcribed Spacer (ITS) and External Transcribed Spacer (ETS) regions derived from the molecular phylogenetic study of Fehlberg and Ranker (2007), with the addition of *Enceliabalandra* (bold and inset photograph). Bootstrap values along branches indicate statistical support for relationships and were generated using 1000 bootstrap replicates in RAxML using a GTRCAT molecular substitution model (Stamatakis 2014).

To understand phylogenetic affinities of *E.balandra*, we used the Internal Transcribed Spacer (ITS) and External Transcribed Spacer (ETS) of nuclear ribosomal DNA. Both spacers are commonly employed in fine-scale studies of angiosperm relationships and this region has been used for understanding relationships amongst closely-related species within Enceliinae (Fehlberg and Ranker 2007). We extracted DNA and amplified both regions from leaf material sampled from the type specimen. When compared with publicly available DNA sequence data using the BLAST algorithm, our sequences for ITS and ETS were matched to members of *Encelia* with high percentage identity (ITS: 98.3% match with *Enceliafrutescens* A. Gray MF963851.1; ETS: 98.34% match with Enceliafarinosavar.farinosa A. Gray DQ383844). The ML phylogeny output from RaxML placed our study species within the genus *Encelia* with high support, confirming conclusions drawn from morphological evidence as shown in Fig. 4. Within *Encelia*, *E.balandra* occupies an early diverging branch of the frutescens clade with moderate bootstrap support.

#### GenBank accession numbers.

ITSMZ892906, ETSMZ576216.

## ﻿Discussion

With the addition of this new species, *Encelia* includes 16 species and five varieties (Clark 1998) notable for their eco-phenotypic variation across divergent desert microhabitats (Singhal et al. 2021). *E.balandra* represents the first species of *Encelia* endemic to the Cape region of Baja California Sur, an important biodiversity hotspot with heightened plant endemism (Rebman et al. 2016). As an endemic to a relatively frost-free yet arid climate, *E.balandra* may prove to be an important subject for eco-physiological studies. *Encelia* are interfertile and hybrids form with relative ease (Clark 1998), though species boundaries are maintained by strong post-dispersal selection influenced by environmental factors (Kyhos et al. 1981; DiVittorio et al. 2020). The possibility that *E.balandra* may be of hybrid origin is also, therefore, an important hypothesis for future studies to test.

The limited DNA sequence data included in the current study resolves *E.balandra* as a sister lineage to the *Frutescens* clade with moderate statistical support (bs = 80). Morphological affinities between *E.balandra* and the *Frutescens* clade include erect fruiting heads and multicellular strigose trichomes (Clark 1998). Weak affinities to either of the major subclades of *Encelia* suggests that *E.balandra* may fill a Darwinian deficit in our understanding of the biogeographic and evolutionary history of *Encelia*. We recommend its inclusion in broad scale studies of this genus using denser sampling of genomic data and suggest that more intensive botanical surveys in the Cape region are needed for the description and conservation of its unique flora rich in micro-endemics.

Finally, we present a dichotomous key, considering morphological data (Table 1) compiled from taxonomic treatments and floras (Blake 1913; Shreve and Wiggins 1964; Wiggins 1980; Clark 1998). The geographical distribution for three *Encelia* taxa found in the Cape region and that of Magdalena Bay, are illustrated in Fig. 1.

**Table 1.** Comparison of morphological traits of *Enceliabalandra* with other *Encelia* of southern Baja California. (data from: Blake 1913, Shreve and Wiggins 1964, Wiggins 1980 and Clark 1998).

Character/ Taxon	*Enceliabalandra*	*Enceliaconspersa*	Enceliafarinosavar.radians	Enceliafarinosavar.phenicodonta	*Enceliapalmeri*
Size (cm)	< 60 cm tall	< 120 cm tall	< 170 cm tall	< 170 cm tall	< 120 cm tall
Growth habit	Suffruticose perennial	Suffruticose perennial	Woody shrub	Woody shrub	Suffruticose perennial
Receptacle dimensions (height × diameter [mm])	4–6 × 13–15	4–5 × 10–12	2 × 3–4	2 × 3–4	8–10 × 10–20
Leaf					
Texture	Strigose	Hispid to pubescent, canescent	Glabrate	Silvery tomentose	Hispid canescent
Trichome length (mm)	1	1	-	< 1	< 1
Lamina shape	Ovate lanceolate	Obovate	Lanceolate to ovate	Lanceolate to ovate	Broadly ovate
Lamina size (length × width [cm])	3 × 1.5	3.5 × 2.5	8 × 4	7 × 4	3.5–4 × 3.5
Margin	Crenate and lobate	Entire	Dentate and undulate	Entire and undulate	Sparsely dentate
Petiole length (mm)	2–3	4–10	10–40	10–40	3–10
Peduncle length (cm)	20–25	8–12	15–25	15–25	6–8
Capitulum position	Terminal	Terminal and axillary	Terminal	Terminal	Terminal and axillary
Capitula number	Solitary	1(2)	5–10	5–10	2–5
Phyllary shape	Lanceolate to ovate-lanceolate	Linear lanceolate to narrowly ovate	Lanceolate (outer) to ovate-lanceolate (inner)	Lanceolate (outer) to ovate-lanceolate (inner)	Linear to lanceolate
Ray floret number	10–12	12–14	11–18	11–18	14–16(–18)
Ray floret dimensions (length × width [mm])	10 × 6	7–10 × 6	7–11 × 6	7–11 × 6	8–10 × 6
Cypsela					
Shape	Obovoid	Linear-lanceolate	Obovate and emarginate	Obovate and emarginate	Obovate
Dimensions (length × width [mm])	5 × 4	3 × < 1	4.5 × 1	4.5 × 1	4.5 × 1
Texture	Faces glabrous when mature on margin and sides.	Villous marginally, faces pubescent	Margins villous with silky hairs	Margins villous with silky hairs	Villous marginally and sparsely on faces

**Table:** 

1	Flowering panicle 1–2-branched, each branch monocephalous	**2**
–	Flowering panicle 2–4-branched, each up to 5–10 capitul	**3**
2	Leaf and peduncle indumentum hispid, with canescent hairs	***Enceliaconspersa***
–	Leaf and peduncle indumentum strigose, with conspicuous trichomes	***Enceliabalandra***
3	Leaves typically broadly ovate; ray florets 14–18(–20)	***Enceliapalmeri***
–	Leaves typically lanceolate; ray florets 10–12(–14)	**4**
4	Disc floret corollas blackish; leaf blades glabrate and green; phyllaries glabrate	**Enceliafarinosavar.radians**
–	Disc floret corollas purplish; leaf blades persistently white farinose; phyllaries puberulent	**Enceliafarinosavar.phenicodonta**

## Supplementary Material

XML Treatment for Encelia balandra
